# The Fallibility of Scientific Evidence in Medical Jurisprudence

**Published:** 1901-11

**Authors:** T. S. Hopkins

**Affiliations:** Thomasville, Ga.


### THE FALLIBILITY OF SCIENTIFIC EVIDENCE IN MEDICAL JURISPRUDENCE.

By T. S. HOPKINS, M.D.,
THOMASVILLE, GA.

I have recently found in a drawer in my office an old note-book in which, begun nearly fifty years ago and continued for many years, I kept a record of new remedies in disease, their therapeutic action and *modus operandi*, and reports of trials in medical jurisprudence. From this old book I glean the following which illustrates the fallibility of science in detecting poisons, and should be a warning to physicians, who are so often called upon to decide the question of life and death in criminal trials.

Madame LaFarge was tried in Paris for the murder of her husband with arsenic. The stomach of the deceased and its contents was boiled in distilled water and the liquor treated with sulphurated hydrogen, yielding a yellow precipitate. This was submitted to the celebrated Orfila, who declared it no proof unless in the precipitate, on being analyzed, metallic arsenic should be found.

The court then appointed M. DuBois and his son and M. DuPuytren to make further analyses. They with the Marsh test and others found not a trace of arsenic. The prosecution not yet satisfied, caused the body of LaFarge to be exhumed for further analysis. It had been buried seven months. It was brought into court in a state of hideous paste-like decomposition, and the chemists in the presence of the court and jury performed their task.

Orfila, DeVergie and Chevellier were the chemists. The experiment completed Orfila addressed the court thus: "I shall divide what I have to say into four heads."

First, "I shall show that there is metallic arsenic in the body of M. LaFarge."

Second, "That this arsenic does not come from the reagents we have used nor from the earth in which the body has been interred."

Third, "That it is not the arsenic normally present in the human frame;" and,

Fourth, "That it is not impossible to explain the inconsistencies in the evidence of the other experts."

The jury found Madame LaFarge guilty with extenuating circumstances and she was sentenced to imprisonment at hard labor for life. The extenuating circumstances saved her from the guillotine. Two years after the trial of Madame LaFarge, Madame LeCoste was tried for the murder of her husband with arsenic. The LaFarge case was viewed as a precedent upon which to establish the guilt of Madame LeCoste.

M. Chevellier, who assisted Orfila in the analysis in the LaFarge case was present and one of the jury asked him if the quantity of arsenic found by him in the body of LeCoste was equal to that which served as a ground for conviction in the LaFarge case. His reply was, "What was said to be the poison found in the body of LaFarge was imponderable. It was so infinitesimal that it could not fulfil the conditions of a standard of comparison when we use the words more or less." This answer acquitted Madame LeCoste, though the facts in the case presented the strongest evidence of guilt. It is now admitted that when M. DuBois and Dupuytren tested for arsenic in the LaFarge case and found none their reagents were pure. When Orfila tested he supposed his reagents were brought from his laboratory, and of course pure, but he afterwards discovered that they had been purchased by one of his assistants in Paris, and upon examination found to contain arsenic. His report of this discovery procured pardon for Madame LaFarge.

In this case the great scientist injected into the decomposed corpse of LaFarge, seven months after his death and burial, a poison which resulted in the conviction and sentence of his innocent wife. The amount of arsenic in the reagent was very small, but sufficient to prove its presence with the Marsh test.

A man by the name of O'Reiley was tried for murder in Dublin, Ireland, prussic acid said to be the poison used. Christison, the great English toxicologist, was engaged for the defense, but was so long delayed by adverse winds on the passage from Liverpool that the trial was so nearly completed that the judge was charging the jury, the charge adverse to the prisoner, when he entered the court room. Under the circumstances, in justice to the accused, whose life was so fearfully jeopardized, the case was reopened. Christison read the evidence and discovered the reagents used by the prosecution for detecting prussic acid. Then taking from his vest pocket a small glass plate, handed it to the judge with the request that he would "empty" into it a portion of the saliva from his mouth. The judge complied and Christison proceeded to apply the same order of tests which had been used by the Crown's experts for the detection of the suspected prussic acid in the dead body, when to the amazement of the judge, counsel and jury, he demonstrated precisely the same results from this saliva that the tests applied by the prosecution had produced in demonstrating the presence of prussic acid in the body of the deceased. O'Reiley was acquitted.

John Stauff was tried at Darmstadt for the murder of Countess Gorlitz, with diacetate of copper. The only evidence, inconclusive and deficient, was the scientific evidence of the Baron Leibig and another equally distinguished chemist, who by their opposite opinions demonstrated the fallibility of their science. The one insisted that diacetate of copper was harmless after being subjected to ebulition, while the other maintained that its strength was greatly increased and intensified thereby. Stauff was acquitted. Mrs. E. G. Wharton was tried in Baltimore charged with destroying the life of General W. S. Ketchum, of the United States Army, with Tartar emetic. The experts for the prosecution maintained that they had discovered antimony—in that an acidulated solution of the contents of the stomach, when sulphurated hydrogen passed through it gave an orange-red precipitate. Dr. McCullough, for the defense, learned that General Ketchum had taken just before he died, the tincture of yellow jasmine. He prepared a mixture containing the tincture of yellow jasmine, passed through it sulphurated hydrogen, and the tests for the poison and the medicine taken produced identical results, and Mrs. Wharton was acquitted. Dr. McCullough made the test in the presence of the court and jury and saved Mrs. Wharton's life.

In the O'Riley case there was a physiological phenomenon exhibited that hydrocyanic acid, or something so near in chemical proportion or combination as to render it analogous to the poison, formed in the secretions from the mucous membrane of the mouth.

Dr. Sternberg, in his valuable book on Bacteriology, tells us that on a certain occasion in his laboratory in New Orleans, engaged in bacteriological investigations, he concluded to experiment on some rabbits. He injected hypodermically into a number of rabbits eight drops of his saliva, and in from twenty-four to thirty-six hours all so treated died from septicemia.

I do not know whether the doctor got the suggestion from the O'Riley case or not, but I candidly admit that I got the suggestion from him, and in every instance here in my office at Thomasville, Georgia, when like experiments have been made, the results corroborated the report of his experiments, seemingly demonstrating the fact that the human saliva contains at times a toxic element destructive to animal life. The length of time the rabbits survived the introduction of the saliva excludes hydrocyanic acid as the toxic element, but that it did contain a toxic element which when injected into the system caused blood-poison is evidenced by death from septicemia.

Charron and Mousur, of the laboratory of experimental medicine in the College of Paris, report repeated experiments with saliva of the horse, the dog, the cow and man. They report that a few drops of a filtrate from a solution of the saliva from any and all of these animals injected into the ear of the rabbit causes death in from one to three minutes. The almost instantaneous death of the rabbit indicates the presence of the hydrocyanic acid in the saliva; but they attribute it to a substance which causes coagulation of the blood and arrest of the circulation due to the formation of clots in the nerve centers.

According to the dictum of distinguished chemists, the human system is rarely ever free from the presence of different poisons. M. Welcheron, one of the directors of the mines in the Duchy of Baden, shows the universal presence of arsenic in Chalybeate waters, ferruginous earth and clay through which the drinking water percolates, and may occur under circumstances when its presence would not be suspected and might affect the chemical tests employed in medical jurisprudence.

M. DeChamps, in a paper sent to the Academy of Science at Paris, maintains that copper exists normally in the human blood, and this physiological copper should be taken into consideration in all medico-legal investigations.

The vegetables derive the copper from the soil, animals derive it from the plants they eat, and man, who feeds on both, in this way gets it into his system. In like manner with mineral waters may the presence of arsenic be accounted for.

In all the trials reported there was but one conviction, and that an accidental one. In no other case was there any proof of innocence, but the contradictory evidence of "scientific experts" created doubts in the minds of the juries, the benefit of which was given to the accused and acquitted them. The witness chair in Medical Jurisprudence is a dangerous one to occupy. Yet physicians, panoplied in self-conceit, often seek it in pursuit of fame, and when leaving it after a scrutinizing examination by such an erudite and accomplished lawyer as the Hon. W. M. Hammond, Thomasville, Georgia, will realize the fact that they have greatly overestimated their scientific ability, and, in search of fame, found only humiliation and shame.

This is an interesting and important question, worthy not only of consideration, but diligent, persevering investigation, for its solution.

---

### THE JOHN W. GARRETT INTERNATIONAL FELLOWSHIP.

A fellowship has been founded by William Johnston in connection with University College, Liverpool, in memory of the late John W. Garrett, of Baltimore, with the title of the "John W. Garrett International Fellowship in Pathology and Physiology."

